# Is There a Role of Epigenetically Inherited Neurogenesis on Infantile Amnesia? Commentary: Intergenerational Transmission of the Positive Effects of Physical Exercise on Brain and Cognition

**DOI:** 10.3389/fnins.2019.01079

**Published:** 2019-10-11

**Authors:** Alonso Martínez-Canabal, Grecia López-Oropeza, Pilar Duran

**Affiliations:** Department of Cell Biology, Faculty of Sciences, National Autonomous University of México, Mexico City, Mexico

**Keywords:** infantile amnesia, dentate gyrus, hippocampus, exercise, epigenetics, neurogenesis

Infantile amnesia, a failure in early childhood to retain episodic memory for long periods, is a paradoxical phenomenon widely accepted as an everyday life fact, but its causes are extensively ignored. Recently, Josselyn and Frankland (2012) proposed a biological hypothesis which proposes that high rates of postnatal hippocampal neurogenesis are the cause of the failure to retain infant memories. If the appearance of Infantile amnesia is a consequence of high levels of neurogenesis, and according to the recent study of McGreevy et al. (2019) there is an inheritance of basal neurogenesis levels, then, the extent of infantile amnesia might be inherited by an epigenetic mechanism.

After Freud and Brill (1914) mentioned the infantile amnesia, some have appeared trying to explain its origin. Howe and Courage (1993) propose that the cause of Infantile amnesia is the lack of self-consciousness at early ages, while Simcock and Hayne (2002) propose that the absence of a complex language disables the generation of a self-narrative. Therefore, according to these hypotheses, infantile amnesia would be an emergent human characteristic, but comparative studies showed that infantile amnesia is present in many mammals as rats or mice (Campbell et al., 1974). These comparative findings suggest that an integrative biological hypothesis to explain infantile amnesia is needed. A recent biological hypothesis suggests that a switch in maturation between NMDA subunits causes a memory retrieval failure, instead of a complete memory erasure (Travaglia et al., 2016). However, the memory engram activation only partially rescues infantile amnesia; then, the phenomenon is a combination of memory degradation and a retrieval failure (Guskjolen et al., 2018). Interestingly NMDA receptors blockade does not disrupt neurogenesis; therefore, both mechanisms that promote infant memory failure would act independently from one another (Tronel et al., 2010).

Recently, Akers et al. (2014) probed the neurogenic hypothesis of Josselyn and Frankland (2012) showing that elevated neurogenesis in infantile mice is partially responsible for infantile amnesia rescuing episodic-like memories ablating neurogenesis. A massive generation of new mossy fibers from the dentate gyrus to hippocampus-CA3 modifies, or displaces previous synaptic contacts (Toni et al., 2008) and might degrade previously acquired episodic-like memories. The recent study of McGreevy et al. (2019) shows that exercised male mice sire offspring with high rates of hippocampal neurogenesis Although, such high levels of neurogenesis occur in adult mice, normally the rates of proliferation decay with age (Lagace et al., 2007), an effect possibly linked to a decrease and senescence of the pool of neural stem cells (Cameron and McKay, 1999). Then, infantile and juvenile individuals might probably have higher neurogenesis rates than the offspring of sedentary parents. Likewise, they show an increase in hippocampal mitochondrial activity and the activation of certain transcription machinery genes, which could regulate the expression of proliferation-associated proteins. Therefore, given that high rates of neurogenesis are present in litters of runner parents (McGreevy et al., 2019), this phenomenon is probably present from infantile stages, in which case, it might increase infantile amnesia.

The study of McGreevy et al. (2019) shows that aerobic exercise in male parents is sufficient to increase the population of immature hippocampal neurons on the first generation. An example of this population enlargement is the increment of the PH3+ cells, a population of cells in replication which are upregulated in response to aerobic exercise (Llorens-Martin et al., 2010) and the increase in the population of Doublecortin+ immature neuron population (specially in the first stages of neuron maturation which are Calretinin negative). No differences are reported in the population of neural stem cells, and more studies are needed to clarify their dynamics. Furthermore, the first-generation mice perform better in a difficult version of the novel object recognition and pattern recognition tasks, which evaluates the episodic-like memories (Sahay et al., 2011). Other studies showed that offspring of exercised parents show resilience to anxiety and fear recovery after extinction, effects probably associated to the sperm of exercised parents carrying unique noncoding microRNAs and tRNAs (Short et al., 2017), this relates with reports of elevated neurogenesis conferring resilience to anxiety and fear recovery after extinction (Cameron and Glover, 2015; Martínez-Canabal et al., 2019).

Low levels of postnatal neurogenesis can also occur; maternal stress (Belnoue et al., 2013) or abuse substances during pregnancy decrease postnatal neurogenesis (Wang and Gondre-Lewis, 2013); conversely, running or swimming during pregnancy increases neurogenesis in the offspring (Bick-Sander et al., 2006; Lee et al., 2006). Hence, the offspring of stressed dams might show higher memory retention and lower neurogenesis; contrarywise, the offspring of exercised dams would show higher neurogenesis and less memory retention at infantile stages. Then, there is a need for new studies to determine if stressors applied to parents before pregnancy affect newborn neurons in the first generation and the consequences of such manipulations on offspring's cognition (Figure 1).

**Figure 1:**
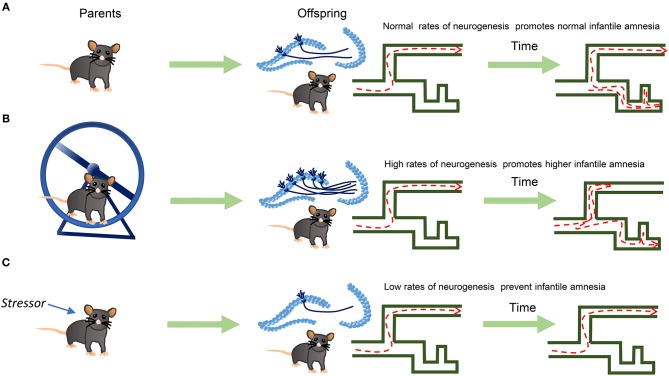
Epigenetic inheritance of newborn neurons and Infantile Amnesia. Parents in aerobic exercise can transmit to the offspring high rates of neurogenesis. Through this mechanism, lower rates of memory retention, or stronger infantile amnesia, may appear in the offspring. **(A)** Parents with no aerobic exercise generate offspring with normal levels of neurogenesis and standard Infantile Amnesia. **(B)** Parents with aerobic exercise, generate offspring with higher levels of neurogenesis and Infantile Amnesia. **(C)** Parents exposed to some stressor show lower levels of neurogenesis than control and therefore more memory retention.

Even though higher rates of memory retention in young ages appear to be an adaptive advantage, hippocampal neurogenesis and infantile amnesia are widespread among mammals and are probably adaptive *per se*. Infantile amnesia possibly allows the retention of only more critical memories, filtering those with a robust emotional component (Richardson et al., 1986) thus retaining only the information that is crucial for the newborn animals' survival. Therefore, parents' physical and mental health can be a critical heritable trait for the offspring. Interestingly, a possible mechanism of this hereditable trait could be microRNAs derived from exercised male parents; which have proved to be critical for hippocampal LTP resulting in cognitive improvement (Short et al., 2017; Benito et al., 2018; McGreevy et al., 2019). Some encouraging advances are identifying that microRNAs correct processing could be related to neurogenesis regulation (Choi et al., 2019), however, a possible link between heritable microRNAs and neurogenesis is not known yet and should be a further research question together with other heritable epigenetic traits.

Growing evidence suggests that hippocampal neurogenesis is critical for spatiotemporal contextualization and emotional responses associated with memory processing even in humans (See references in Kempermann et al., 2018). Defining the epigenetic mechanism of neurogenesis enhancement or downregulation through generations is critical to clarify how the continuous growth of new hippocampal cells regulates, in a transgenerational fashion, the cognition and psychopathologies associated to the hippocampus, like stress or depression.

## Author Contributions

AM-C proposed the idea. AM-C, GL-O, and PD wrote the paper. GL-O prepared the figure.

### Conflict of Interest

The authors declare that the research was conducted in the absence of any commercial or financial relationships that could be construed as a potential conflict of interest.
